# Supplementary material for: Racial differences in people living with HIV and Heart Failure: Insight from New York City health and hospitals HIV Heart Failure Cohort
Source: PLoS One. 2026 Mar 9;21(3):e0343710. doi: 10.1371/journal.pone.0343710 (PMC12970931; doi:10.1371/journal.pone.0343710)
Supplement: S1 Table — (DOCX) [file pone.0343710.s001.docx]

**Supplementary table 1.** Race and Overall Mortality through Cox regression hazard model

| **All-cause mortality** | **HR (95% CI)** | **P value** |
| --- | --- | --- |
| Asian/Pacific islander | 1 |  |
| Black | 1.83 (0.45-7.51) | 0.40 |
| Hispanic/Latino | 2.12 (0.51- 8.83) | 0.30 |
| Non-Hispanic White | 3.23 (0.74- 14.17) | 0.12 |
| Other/Unknown | 4.63 (1.07-20) | 0.04 |

Model adjusted for age, sex, baseline EF, controlled HIV, and comorbidities such as chronic obstructive pulmonary disease (COPD), end-stage renal disease (ESRD), cancer, hyperlipidemia, hypertension, diabetes mellitus, peripheral artery disease (PAD), pulmonary hypertension, and coronary artery disease (CAD), ADL and smoking status
