# Supplementary material for: Racial differences in people living with HIV and Heart Failure: Insight from New York City health and hospitals HIV Heart Failure Cohort
Source: PLoS One. 2026 Mar 9;21(3):e0343710. doi: 10.1371/journal.pone.0343710 (PMC12970931; doi:10.1371/journal.pone.0343710)
Supplement: S2 Table — (DOCX) [file pone.0343710.s002.docx]

**Supplementary table 2.** Potential effect modifier with race to mortality

| **Controlled HIV as modifier** | **HR** | **95% CI** | **P-value** |
| --- | --- | --- | --- |
| Controlled HIV x non-Hispanic White | **0.51** | **0.14-1.86** | **0.31** |
| Controlled HIV x Black | **0.65** | **0.24-1.77** | **0.40** |
| Controlled HIV x Hispanic/Latino | **0.83** | **0.29-2.45** | **0.75** |
| Controlled HIV x Asian/Pacific Islander | **0.25** | **0.01-4.72** | **0.36** |
| Controlled HIV x Other/known | **Unable to calculate given limited case** | |  |
