# Supplementary material for: Racial differences in people living with HIV and Heart Failure: Insight from New York City health and hospitals HIV Heart Failure Cohort
Source: PLoS One. 2026 Mar 9;21(3):e0343710. doi: 10.1371/journal.pone.0343710 (PMC12970931; doi:10.1371/journal.pone.0343710)
Supplement: S3 Table — (DOCX) [file pone.0343710.s003.docx]

**Supplementary table 3.** Sensitivity analysis within heart failure with reduced EF patient

| **All-cause mortality in heart failure with reduced EF** | **HR (95% CI)** | **P value** |
| --- | --- | --- |
| Asian/Pacific islander | 1 |  |
| Black | 1.53 (0.36, 6.59) | 0.57 |
| Hispanic/Latino | 1.58 (0.36, 6.98) | 0.54 |
| Non-Hispanic White | 1.88 (0.37, 9.42) | 0.44 |
| Other/Unknown | 4.43 (0.91, 21.56) | 0.07 |
| **All-cause mortality in controlled HIV** |  |  |
| Asian/Pacific islander | 1 |  |
| Black | 2.11 (0.29, 15.58) | 0.47 |
| Hispanic/Latino | 2.59 (0.35, 19.40) | 0.36 |
| Non-Hispanic White | 3.20 (0.39, 25.95) | 0.28 |
| Other/Unknown | 5.69 (0.73, 44.51) | 0.09 |

Model adjusted for age, sex, baseline EF, controlled HIV, and comorbidities such as chronic obstructive pulmonary disease (COPD), end-stage renal disease (ESRD), cancer, hyperlipidemia, hypertension, diabetes mellitus, peripheral artery disease (PAD), pulmonary hypertension, and coronary artery disease (CAD), ADL and smoking status
