# Supplementary figures and images for: Racial differences in people living with HIV and Heart Failure: Insight from New York City health and hospitals HIV Heart Failure Cohort
Source: PLoS One. 2026 Mar 9;21(3):e0343710. doi: 10.1371/journal.pone.0343710 (PMC12970931; doi:10.1371/journal.pone.0343710)

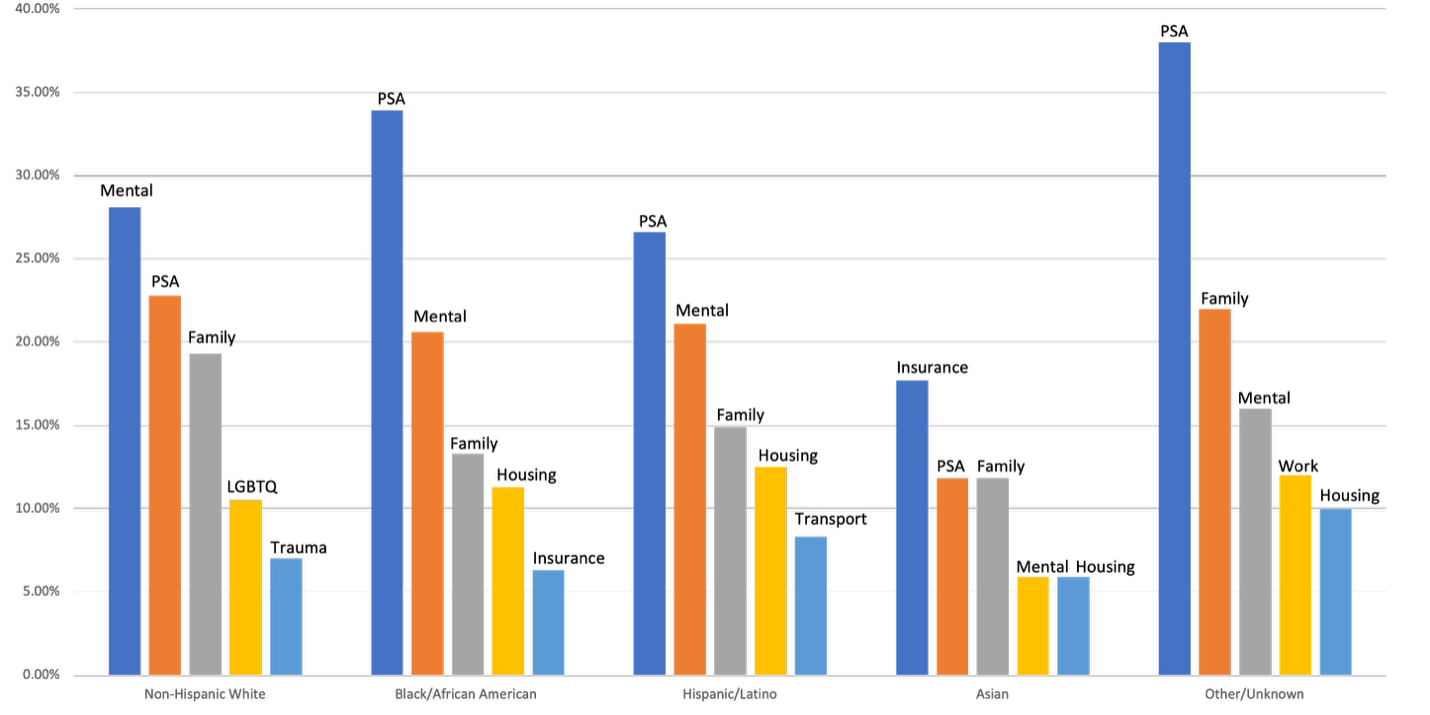

Supplement: S1 Fig — (PNG) [file pone.0343710.s004.png]
